# Significantly Decreased Islet *β* Cell Function is Closely Associated with Hyperglycemia in Chronic Hepatitis B Patients

**DOI:** 10.1155/2021/1264707

**Published:** 2021-08-24

**Authors:** Dafeng Liu, Lingyun Zhou, Xinyi Zhang, Yilan Zeng, Lang Bai, Dongbo Wu, Hong Tang

**Affiliations:** ^1^Center of Infectious Diseases, Sichuan University West China Hospital, Chengdu, China; ^2^Department of Internal Medicine, The Public and Health Clinic Centre of Chengdu, Chengdu, China; ^3^Department of Endocrinology and Metabolism, Sichuan University West China Hospital, Chengdu, China

## Abstract

**Aim:**

This study is aimed at the characteristics of glucose metabolism and islet *β* cell function evaluated by the homeostasis model assessment of *β* cell function (HOMA-*β*) value and its risk factors in chronic hepatitis B (CHB) patients.

**Method:**

This cross-sectional study recruited 110 CHB patients (CHB group) and 110 patients without hepatitis B virus (non-HBV group); the groups were matched according to sex, age, and body mass index under the same glucose metabolism status. The risk factors, characteristics, and differences in glucose metabolism and HOMA-*β* values between the two groups were analyzed.

**Results:**

The abnormal glucose metabolism rate was higher in CHB patients with liver cirrhosis (LC) or hepatitis B envelope antigen (HBeAg) (−) status. In addition, under the same glucose metabolism status, the fasting plasma glucose (FPG) levels and 2-hour postprandial plasma glucose (2h-PG) levels in the CHB group were higher, while the HOMA-*β* values were significantly lower and the homeostasis model assessment of insulin resistance (HOMA-IR) value was not higher than that in the non-HBV group (all *P* < 0.0001). Further analyses revealed that the main risk factors for abnormal glucose metabolism were HBeAg (−) status and hepatitis B envelope antibody levels. But HBV serological and virological indicators had no effects on the HOMA-*β* values.

**Conclusion:**

Islet *β* cell function in patients with CHB was compromised, which is closely associated with fasting and postprandial hyperglycemia in chronic hepatitis B patients. Further research should be done to verify the compromised islet *β* cell function and then to investigate the mechanisms behind the effect of hepatitis B virus infection on islet *β* cell function in CHB patients.

## 1. Introduction

According to the WHO report, there are approximately 257 million people with chronic hepatitis B virus (HBV) infections in the world [1]. Approximately 887,000 people die of HBV infection-related diseases annually, of which liver cirrhosis (LC) accounts for 30% and primary hepatocellular carcinoma (HCC) accounts for 45%. In China, among patients with liver cirrhosis and HCC, 77% and 84% of respective cases are caused by HBV.

China is one of the HBV middle- and low-endemic areas worldwide. In 2014, the results of a seroepidemiological survey of hepatitis B among people aged 1 to 29 years by the Chinese Center for Disease Control and Prevention showed that the prevalence of hepatitis B surface antigen (HBsAg) in the populations aged from 1 to 4 years , from 5 to 14 years, and from 15 to 29 years was 0.32%, 0.94%, and 4.38%, respectively [2]. It is estimated that the current prevalence of HBsAg in the general population is 5% to 6%, and there are approximately 70 million people with chronic HBV infection, of which approximately 2 million to 3 million patients present with chronic hepatitis B (CHB) [3].

Although the relationship between hepatitis B virus infection and diabetes mellitus (DM) remains controversial [4–6], several studies have shown that the prevalence of DM is significantly higher in the HBV-infected population [4, 7–10], particularly in those with high viral load, with a long duration of CHB [7], with cirrhosis [4–8], of Asian American race [9], or of non-Asian ethnicity with long-term residence in North America [10].

DM can promote the progression of liver fibrosis and cirrhosis [11–15]. For DM patients with cirrhosis, the leading cause of death is hepatic failure as opposed to complications of DM. Furthermore, DM may also promote HCC and lead to poorer prognosis after liver transplantation [16, 17]. Therefore, the coexistence of abnormal glucose metabolism and IR could promote the progression and worsen the prognosis of CHB.

Impaired glucose regulation and diabetes mellitus mostly manifest as high postprandial glucose levels in plasma and are commonly associated with IR or islet *β* cell dysfunction or both in the general population. The characteristics of glucose metabolism, abnormal glucose metabolism due to IR or islet *β* cell dysfunction, the characteristics of islet *β* cell function as indicated by the homeostasis model assessment of *β* cell function (HOMA-*β*) value [18], and its risk factors in chronic hepatitis B (CHB) patients are unclear. This study is aimed at the characteristics of glucose metabolism and islet *β* cell function evaluated by HOMA-*β* value and its risk factors in CHB patients.

## 2. Methods

### 2.1. Subjects

A cross-sectional study with a sample size of 220 patients was conducted in the Public and Health Clinic Centre of Chengdu from January 1, 2019, to June 30, 2020 [19, 20]. Among these subjects, 110 patients with CHB were entered into the CHB group. 110 patients without hepatitis B virus, hepatitis C virus (HCV), and human immunodeficiency virus (HIV) infection who were matched to the CHB group according to sex, age, and body mass index (BMI) under the same glucose metabolism status were entered into the non-HBV group. The study was approved by the ethics committee of the Public and Health Clinic Centre of Chengdu (PJ-K2019-019-01). All patients provided written informed consent.

### 2.2. The Inclusion Criteria and the Selection Criteria

The inclusion criteria for the CHB group were as follows: (1) outpatients or inpatients with CHB or post-hepatitis B cirrhosis, (2) individuals who agreed to undergo noninvasive ultrasound liver stiffness measurement, and (3) individuals aged 18–70 years.

The selection criteria of the non-HBV group were as follows: patients without hepatitis B virus, hepatitis C virus (HCV), and human immunodeficiency virus (HIV) infection who were matched to the CHB group according to sex, age, and body mass index (BMI) under the same glucose metabolism status.

The following exclusion criteria were used in this study: (1) other hepatitis virus or human immunodeficiency virus infections; (2) hepatocellular carcinoma; (3) ascites; (4) decompensated cirrhosis; (5) hepatic function alanine aminotransferase (ALT) or aspartate aminotransferase (AST) level higher than the 5-fold upper limit of the normal value 37 IU/L, total bilirubin level higher than the 2-fold upper limit of the normal value 17.1 *μ*mol/L within the last 6 months, or prothrombin activity (PT%) <60%; and (6) BMI >30 kg/m^2^ or <18.5 kg/m^2^.

### 2.3. The Diagnostic Criteria

The diagnostic criteria of the diseases were as follows: CHB diagnostic and typing criteria and impaired glucose regulation (IGR) and DM diagnostic criteria were applied according to the corresponding guidelines [21, 22].

### 2.4. Grouping Standards

The participants were divided into the three following subgroups according to their glucose metabolism status. Those without prediabetes or diabetes mellitus history had their glucose metabolism status assessed by the 75 g oral glucose tolerance test (OGTT) and glycated hemoglobin A1c test. Glucose metabolism status was as follows: the normal glucose tolerance (NGT) condition group [fasting plasma glucose (FPG) levels for OGTT<6.0 mmol/L, 2-hour postprandial glucose (2h-PG) levels for OGTT <7.8 mmol/L, and glycated hemoglobin A1c (HbA1c) levels <6.0%], the IGR condition group (FPG, 2h-PG and HbA1c levels all between those in the NGT and DM condition), and the DM condition group (FPG levels for OGTT ≥7.0 mmol/L and 2h-PG levels for OGTT ≥11.1 mmol/L, or twice that of the FPG or 2h-PG levels meeting the criteria, or HbA1c ≥6.5%).

**Figure 1:**
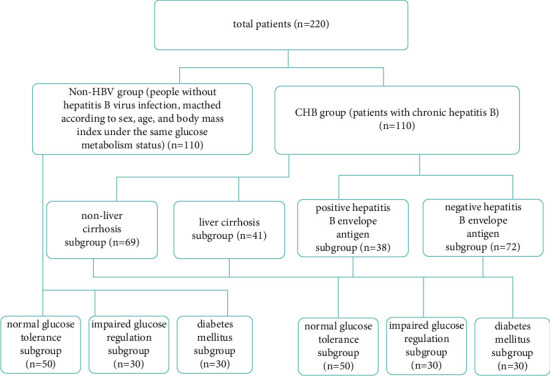
Patient data (*n* = 220). CHB, chronic hepatitis B; HBV, hepatitis B virus; LC, liver cirrhosis; HBeAg (−), hepatitis B envelope antigen negative; HBeAg (+), hepatitis B envelope antigen positive; NGT, normal glucose tolerance; IGR, impaired glucose regulation; DM, diabetes mellitus.

Of the 110 CHB patients or 110 patients without HBV, 50, 30, and 30 patients were divided into the NGT subgroup, the IGR subgroup, and the DM subgroup, respectively (Figure 1).

Of 110 CHB patients, 69 and 41 patients were further divided into the non-LC subgroup (those without LC) and the LC subgroup (those with LC) (Figure 1), and 38 and 72 patients were also further divided into the hepatitis B envelope antigen (HBeAg) (+) subgroup and HBeAg (−) subgroup (Figure 1).

### 2.5. Data Collection

Demographic information (age and sex), anthropometric parameters (body weight and height), and glucose metabolic parameters [FPG levels, 2h-PG levels, fasting insulin (FINS) levels, 2-hour postprandial insulin (2h-INS) levels, and hemoglobin A1c (HbA1c) levels] were obtained. BMI, HOMA-IR values, and HOMA-*β* values were calculated by using the following formulas: BMI = weight (kg)/height (m^2^), HOMA-IR = FINS (Um/L) × FPG (mmol/L)/22.5, and HOMA-*β* = 20 × FINS (Um/L)/[FPG (mmol/L) − 3.5] [18].

Databases were established according to the research needs. Two researchers simultaneously collected and entered the data into the database. Then, the researchers checked all of the data for assessment to ensure data integrity, authenticity, and accuracy.

### 2.6. Statistical Analysis

The Statistical Package for the Social Sciences software version 17.0 (IBM Inc., Armonk, NY, USA) and Prism Version 8 (GraphPad Inc., US) were used for statistical analysis. Age, BMI, FPG, and FINS levels and HOMA-IR values had a normal distribution, and the statistical analysis was conducted directly. Natural HOMA-*β* values were logarithmically transformed before the statistical analysis. The measurement data are expressed as *x* ± SD, ANOVA was used for a multigroup comparison with variance homogeneity and normal distribution data, and the least significant difference (LSD) *t*-test was used for further comparison between the two groups. When the data did not have homogeneity of variance and a normal distribution, an independent-sample Kruskal-Wallis H (K) test was used for multigroup comparisons, while a Mann-Whitney *U* test was used for further comparisons between two groups. An independent-sample *t*-test was used to make comparisons between two groups. A percentage or proportion was used to express enumeration data, and a chi-square test was used for comparisons of these data. Spearman correlation analysis was used for the two-factor correlation analysis, and multiple stepwise regression was used for the multifactor correlation analysis. Statistical significance was defined as *P* < 0.05.

## 3. Results

### 3.1. Baseline Conditions

No significant differences were observed in terms of age, sex, BMI, or glucose metabolism status between the two groups (Table 1). For the CHB group, 41 (37.27%) patients had LC, and 38 (34.55%) patients were HBeAg (+). In the non-HBV group, there were no cases of LC.

### 3.2. The Prevalence of Abnormal Glucose Metabolism

The IGR and DM rates in the CHB group were all 27.27% (30/110). In addition, the abnormal glucose metabolism rate (including IGR and DM) in the LC subgroup was significantly higher than that in the non-LC subgroup (Table 2), and the corresponding rate in the HBeAg (−) subgroup was also significantly higher than that in the HBeAg (+) subgroup (Table 3).

### 3.3. Fasting and Postprandial Hyperglycemia in CHB Patients

**Figure 2:**
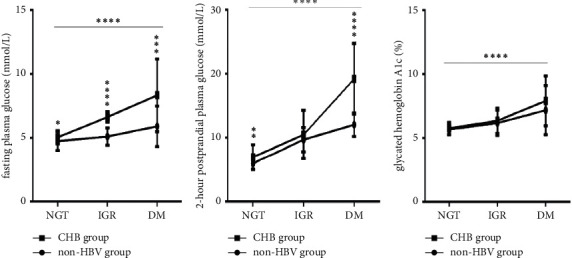
Comparison of the glucose metabolism parameters between the two groups under NGT, IGR, and DM conditions (*n* = 220; NGT, IGR, and DM in the two groups, *n* = 50, 30, and 30, respectively). (a) FPG levels; (b) 2h-PG levels; (c) HbA1c. FPG, fasting plasma glucose; 2h-PG, 2-hour postprandial glucose; HbA1c, glycated hemoglobin A1c; NGT, normal glucose tolerance; IGR, impaired glucose regulation; DM, diabetes mellitus; CHB, chronic hepatitis B; HBV, hepatitis B virus. Two-way ANOVA was used for the interaction comparison ((a)–(c), all *P* < 0.0001). One-way ANOVA was used for intragroup comparisons ((a)–(c), all *P* < 0.0001). Unmatched *t*-tests were used for the intergroup comparisons. ^*∗*^*P* < 0.05, ^*∗∗*^*P* < 0.01, ^*∗∗∗*^*P* < 0.001, and ^*∗∗∗∗*^*P* < 0.0001.

With the deterioration of glucose metabolism from NGT and IGR to DM, FPG levels, 2-hour PG levels, and HbA1c levels (Figures 2(a)–2(c)) all continuously increased in the two groups (all *P* < 0.0001).

Under the same glucose metabolism conditions, FPG levels (Figure 2(a)) in the CHB group were always higher than those in the non-HBV group (all *P* < 0.05), especially under IGR and DM conditions (all *P* < 0.0001). Moreover, for the non-HBV group, under IGR and DM conditions, the FPG levels (Figure 2(a)) were all lower than 6.0 mmol/L, while in the CHB group, the FPG levels were more than 6.0 mmol/L under IGR conditions and more than 7.0 mmol/L under DM conditions. Under NGT and DM conditions, 2-hour PG levels (Figure 2(b)) in the CHB group were also higher than those in the non-HBV group (*P* < 0.01 and *P* < 0.0001, respectively). However, under the same glucose metabolism conditions, there was no difference in HbA1c levels (Figure 2(b)) between the two groups (all *P* > 0.05).

### 3.4. Significantly Decreased Islet *β* Cell Function, Not IR, Is Closely Related to Fasting and Postprandial Hyperglycemia in CHB Patients

**Figure 3:**
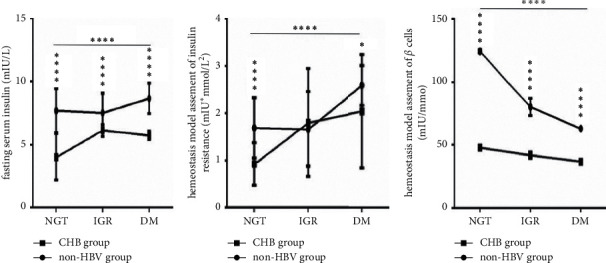
Comparison of the insulin resistance and islet *β* cell function between the two groups under NGT, IGR, and DM conditions (*n* = 220; NGT, IGR, and DM in the two groups, *n* = 50, 30, and 30, respectively). (a) FINS levels; (b) HOMA-IR values; (c) HOMA-*β* values. FINS, fasting serum insulin; HOMA-IR, homeostasis model assessment of insulin resistance; HOMA-*β*, homeostasis model assessment of *β* cell function; NGT, normal glucose tolerance; IGR, impaired glucose regulation; DM, diabetes mellitus; CHB, chronic hepatitis B; HBV, hepatitis B virus. Two-way ANOVA was used for the interaction comparison ((a) and (c), all *P* < 0.0001; (b), *P* < 0.01). One-way ANOVA was used for intragroup comparisons ((a)–(c), all *P* < 0.0001). Unmatched *t*-tests were used for the intergroup comparisons. ^*∗*^*P* < 0.05 and ^*∗∗∗∗*^*P* < 0.0001.

With the deterioration of glucose metabolism from NGT and IGR to DM, both the FINS levels and the HOMA-IR values (Figures 3(a) and 3(b)) continuously increased in the two groups (both *P* < 0.0001), while the HOMA-*β* values (Figure 3(c)) were significantly and continuously decreased in the two groups (*P* < 0.0001). However, under the same glucose metabolism conditions in the CHB group, HOMA-IR values (Figure 3(b)) were not higher, but HOMA-*β* values (Figure 3(c)) were significantly lower than those in the non-HBV group, especially under NGT conditions (all *P* < 0.0001).

### 3.5. Characteristics of Glucose Metabolism in CHB Patients with LC

**Figure 4:**
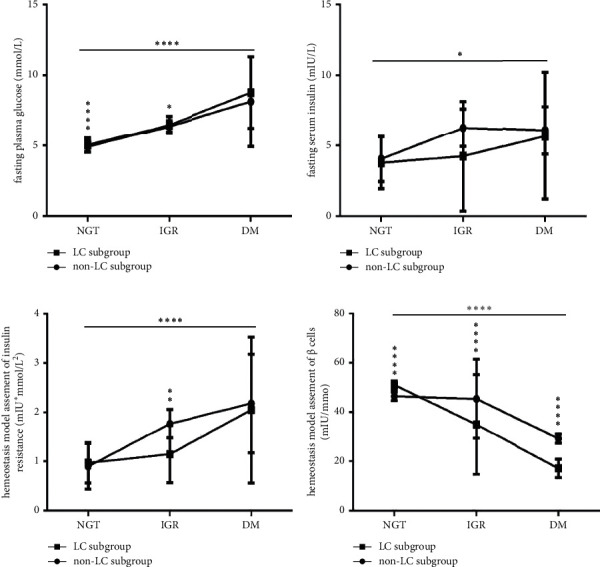
Comparison of glucose metabolism parameters between the non-LC and LC CHB subgroups under NGT, IGR, and DM conditions (*n* = 110; NGT, IGR, and DM in non-LC subgroup, *n* = 42, 12, and 15, respectively; in LC subgroups, *n* = 8, 18, and 15, respectively). (a) FPG levels; (b) FINS levels; (c) HOMA-IR values; (d) HOMA-*β* values. FPG, fasting plasma glucose; FINS, fasting serum insulin; HOMA-IR, homeostasis model assessment of insulin resistance; HOMA-*β*, homeostasis model assessment of *β* cell function; NGT, normal glucose tolerance; IGR, impaired glucose regulation; DM, diabetes mellitus; CHB, chronic hepatitis B; HBV, hepatitis B virus; LC, liver cirrhosis. Two-way ANOVA was used for the interaction comparison ((a), *P* < 0.05; (d), *P* < 0.0001). One-way ANOVA was used for intragroup comparisons ((a), (c), and (d), all *P* < 0.0001; (b), *P* < 0.05). Unmatched *t*-tests were used for the intergroup comparisons. ^*∗*^*P* < 0.05, ^*∗∗*^*P* < 0.01, and ^*∗∗∗*^*P* < 0.001.

With the deterioration of glucose metabolism from NGT and IGR to DM conditions, the FPG levels (Figure 4(a)), HOMA-IR values (Figure 4(c)), and FINS levels (Figure 4(b)) were all continuously increased ((a) and (b), all *P* < 0.0001; (c), *P*=0.0185), while the HOMA-*β* values (Figure 4(d)) were all continuously decreased (*P* < 0.0001) in both the LC and non-LC subgroups.

In the LC subgroup, the HOMA-*β* values (Figure 4(d)) were higher under NGT conditions but significantly lower under IGR and DM conditions than those in the non-LC subgroup (all *P* < 0.0001). At the same time, the FPG levels (Figure 4(a)) were higher under NGT and IGR conditions than in the non-LC subgroup (*P* < 0.0001 and *P* < 0.05, respectively). However, there were no significant differences in the FINS levels (Figure 4(b)) or the HOMA-IR values (Figure 4(c)) under the same glucose metabolism conditions or in the FPG levels under DM conditions between the LC and non-LC subgroups (all *P* > 0.05).

### 3.6. Characteristics of Glucose Metabolism in CHB Patients with HBeAg (−)

**Figure 5:**
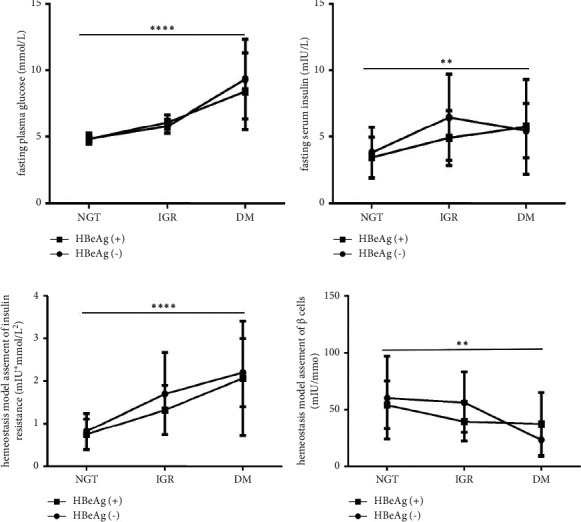
Comparison of glucose metabolism parameters between the HBeAg (+) and HBeAg (−) subgroups under NGT, IGR, and DM conditions (*n* = 110; NGT, IGR, and DM in HBeAg (−) subgroup, *n* = 24, 23, and 25, respectively; in HBeAg (+) subgroup, *n* = 26, 7, and 5, respectively). (a) FPG levels; (b) FINS levels; (c) HOMA-IR values; (d) HOMA-*β* values. FPG, fasting plasma glucose; FINS, fasting serum insulin; HOMA-IR, homeostasis model assessment of insulin resistance; HOMA-*β*, homeostasis model assessment of *β* cell function; NGT, normal glucose tolerance; IGR, impaired glucose regulation; DM, diabetes mellitus; HBeAg (−), hepatitis B envelope antigen negative; HBeAg (+), hepatitis B envelope antigen positive. Two-way ANOVA was used for interaction comparisons ((a)–(d), all *P* > 0.05). One-way ANOVA was used for intragroup comparisons ((a) and (c), all *P* < 0.0001; (b) and (d), all *P* < 0.01). Unmatched *t*-tests were used for the intergroup comparisons ((a)–(d), all *P* > 0.05).

With the deterioration of glucose metabolism from NGT and IGR to DM status, the FPG levels (Figure 5(a)), FINS levels (Figure 5(b)), and HOMA-IR values (Figure 5(c)) of the two subgroups were all continuously increased ((a) and (c) all *P* < 0.0001; (b), *P*=0.0055), while the HOMA-*β* values (Figure 5(d)) were all continuously decreased (*P*=0.0031) in both the HBeAg (+) and HBeAg (−) subgroups.

Similar changes in all glucose parameters (Figures 5(a)–5(d)) could be seen in both the HBeAg (+) and HBeAg (−) subgroups, and there was no significant difference between the two subgroups under the same glucose metabolism conditions (all *P* > 0.05).

### 3.7. The Risk Factors for Abnormal Glucose Metabolism

According to the Spearman correlation analysis, LC, hepatitis B envelope antibody (HBeAb) levels, alkaline phosphatase (ALP) levels, gamma glutamyl transferase (GGT) levels, and liver stiffness measurement (LSM) levels were positively correlated, but HBeAg (+), hepatitis B surface antigen (HBsAg) levels, HBeAg levels, hepatitis B core antibody (HBcAb) levels, and hepatitis B viral nucleic acid (HBV DNA) levels were all negatively correlated with glucose metabolism status (Table 4). In addition, HBeAb levels, ALP levels, GGT levels, and LSM levels were also positively correlated, while HBeAg (+), HBsAg levels, HBeAg levels, and HBV DNA levels were all negatively correlated with FPG levels (Table 4). Based on multiple stepwise regression analysis, HBeAg (−), GGT levels, and HBeAb levels were risk factors for glucose metabolism, and HBeAb levels were risk factors for FPG levels (Table 5).

According to the Spearman correlation analysis, LC and LSM levels were positively correlated with FINS levels; simultaneously, LC, LSM levels, and HBeAb levels were also positively correlated with HOMA-IR values, while hepatitis B surface antibody (HBsAb) levels were negatively correlated with both FINS levels and HOMA-IR values (Table 4). Based on multiple stepwise regression analysis, LC was a risk factor for both FINS levels and HOMA-IR values (Table 5).

Only the HBsAg levels were positively correlated, while the HBsAb levels and ALP levels were negatively correlated with HOMA-*β* values, but only the ALP level was a risk factor for HOMA-*β* values (Table 5).

## 4. Discussion

This study revealed that the prevalence of both IFG and DM was 27.27% in CHB patients, and the abnormal glucose metabolism rate was higher in CHB patients with LC or HBeAg (−) status.

Some of the findings in this study were similar to those in previous literature, which reported that the prevalence of DM is significantly higher in the HBV-infected population [4–11], particularly in individuals with high viral loads, with a long duration of CHB [7], with cirrhosis [4, 5, 7, 8], of Asian-American race [9], or of non-Asian race with long-term residency in North America [10]. A meta-analysis reported that the summary OR of the risk of DM for HBV patients was 1.99 (95% CI, 1.08–3.65) compared with that of non-HBV individuals [4]. The prevalence of both IFG and DM in this study was higher than the 12.5% for DM and 7.8% for IFG in adults with CHB previously reported in a large HBV-infected multiethnic cohort study [9]. This might be attributed to the differences in the study population: the populations of the two former studies were Chinese, while the population of the latter study was American.

This study also reported a higher abnormal glucose metabolism (including IGR and DM) rate in CHB patients with LC. The prevalence of IGR and DM was 43.90% and 36.59% in CHB patients with LC in comparison to 18.42% and 13.16% in CHB patients without LC, respectively. This finding is consistent with those from a study [8–11, 15] in which the odds ratios for DM in chronic hepatitis B cirrhosis patients compared with nonchronic hepatitis B patients were 1.74, 1.76, and 2.317 (95% confidence intervals: 1.43–2.13, 1.44–2.14, and 1.528–3.513, respectively) [8–11, 15]. However, the prevalence of DM was higher than that in another cross-sectional study, which reported prevalence of 22.2% of DM among CHB patients with liver cirrhosis [15]. The development of cirrhosis may increase the incidence of DM [8–11, 15].

In addition, the abnormal glucose metabolism rate was higher in CHB patients who were HBeAg-negative, with IGR and DM prevalences of 31.94% and 34.72% in patients who were HBeAg-negative in comparison to 17.39% and 21.74%, respectively, in patients who were HBeAg-positive. Previous studies suggested that HBsAg status could influence glucose metabolism, and maternal HBsAg carriage was an independent risk factor for gestational diabetes mellitus (GDM) [23]. The incidence of GDM in pregnant women who were HBsAg-positive was 6.48%, which was higher than the 3.41% incidence rate in those who were HBsAg-negative [24]. However, there was no significant association between the incidence of DM and viral load, HBeAg carrier status, or other HBV markers in pregnancy [23, 24]. There is no literature report on the correlation between the incidence of DM and HBeAg carrier status in CHB patients.

Further analyses demonstrated that characteristics of glucose metabolism in patients with CHB manifested as elevated FPG and 2-hour PG levels. We found that, under the same glucose metabolism conditions, the FPG and 2-hour PG levels of the CHB group were continuously higher than those of the non-HBV group; the FPG level was more than 6.0 mmol/L under IGR conditions and more than 7.0 mmol/L under DM conditions, while in the non-HBV group, it was lower than 6.0 mmol/L under all three glucose metabolism conditions.

Increased FPG and 2-hour PG were manifested as significantly decreased islet *β* cell function, as indicated by the HOMA-*β* values, and were not manifested as insulin resistance, as indicated by the HOMA-IR values. Under NGT conditions, the HOMA-*β* value of the HBV group was 47.53 mIU/mmol, that is, only half of the reference value (100.00 mIU/mmol) and only one-third of that of the non-HBV group (124.19 mIU/mmol), and these values continuously decreased with the deterioration of glucose metabolism.

A multicenter randomized parallel-group trial showed that the HOMA-*β* value in patients newly diagnosed with DM was only half the reference value (100 mmol ^*∗*^ mIU/L^2^); it decreased progressively at a rate of 4.5% annually and deteriorated with the course of the disease [25]. A new staging method for NGT, IGR, and DM was proposed according to the function of *β* cells: normal phase of *β* cell function, compensatory phase of *β* cell function, decompensatory phase of *β* cell function, and failure phase of *β* cell function in the general population. The compensatory secretion of *β* cell function occurs in individuals with NGT and IR and reaches the peak of compensatory secretion. The decompensatory phase of *β* cell function happens in individuals with prediabetes or IGR [26]. In recent years, most studies have confirmed that not all individuals with NGT are healthy, and some present with IR [27]. Individuals with NGT who have both IR and dysfunction of *β*-cell have a significantly increased risk of prediabetes and/or DM [27]. Therefore, the HOMA-*β* values of CHB patients under NGT conditions were even lower than those of newly diagnosed patients with DM. The *β* cell function of the CHB population deteriorates directly to the decompensatory and failure phases, without undergoing normal and compensatory phases, even under NGT conditions, and this change leads to higher FPG and 2-hour PG levels and high prevalence of IGR and DM in the CHB population. From this, we can conclude that the evident increase in the FPG and 2-hour PG levels in patients with CHB was associated with worsening *β* cell function but not insulin resistance.

In this study, it was also demonstrated that most of the HBV serological and virological indicators had negative effects, while LC, HBeAb levels, and markers of liver inflammation and fibrosis had positive effects on both glucose metabolism and FPG levels. The main risk factors for glucose metabolism and FPG levels were HBeAg (−) and HBeAb levels. However, HBV serological and virological indicators had no direct effects on islet *β* cell function, as indicated by the HOMA-*β* values. Therefore, we speculated that HBV indirectly affected islet *β* cell function through certain mechanisms.

Fundamental studies have found that hepatitis B virus infection could increase the production of tumor necrosis factor (TNF), especially in HBeAg-negative patients [28]. The overproduction of TNF could decrease the phosphorylation of insulin receptor substrates 1 and 2, inhibit phosphoinositol 3-kinase and protein kinase B, block the phosphorylation of glucose transporter 4, prevent the cell uptake of glucose [29], and increase plasma glucose levels. Prostate six-transmembrane protein 2 (STAMP2) is a factor associated with inflammation and dietary adipocyte function and system metabolism. It can be induced by nutrition, feeding, and cytokines, such as TNF alpha, interleukin (IL)-1*β*, and IL-6, which can inhibit IR in rats. IR and visceral and hepatic insulin signaling disorders were observed in mice lacking STAMP2. In the presence of inflammation and obesity, the increased expression of STAMP2 has protective effects against insulin signaling in the liver [30]. Moreover, hepatitis B virus X protein induces liver fat accumulation and IR by reducing the expression of STAMP2. STAMP2 downregulates the insulin-induced phosphorylation of the P3K p85 subunit, protein kinase, and the expression of insulin receptor substrate 1, and the posttranscriptional level of insulin receptor substrate 1 plays a role [31]; this leads to the increase in blood glucose levels and high abnormal glucose metabolism incidence.

Hyperglycemia can lead to excessive production of reactive oxygen species (ROS) which trigger a variety of molecular mechanisms, such as activation of proinflammatory signaling pathways, increased secretion of proinflammatory cytokines, which in turn leads to chronic systemic inflammation, and activation of cell apoptosis and tissue damage [32, 33]. ROS can impact insulin signal pathways, thus leading to consequent insulin resistance [34, 35]. Moreover, inflammation is one of the underlying risks of *β* cell damage and IR in patients with T2DM [33, 36].

Although those fundamental science studies have confirmed that hepatitis B virus infection could lead to increased hepatic glucose output and IR, they could not explain the decrease in HOMA-*β* values and FINS levels. Whether oxidative stress mediates islet *β* cell dysfunction has not been confirmed by experimental studies. Further fundamental scientific studies are needed to investigate the mechanisms behind the effect of hepatitis B virus infection on islet *β* cell function in CHB patients.

To our knowledge, this cross-sectional study is the first to compare the differences in HOMA-*β* values between CHB patients and non-HBV patients matched according to sex, age, and BMI under the same glucose metabolism status and to investigate the characteristics of islet *β* cell function and its risk factors in CHB patients. The results showed that the *β* cell function of the CHB population deteriorated directly to the decompensatory and failure phases without undergoing normal and compensatory phases, even under NGT conditions. Significantly decreased islet *β* cell function, not IR, was associated with fasting and postprandial hyperglycemia. In addition, HBV could directly affect glucose metabolism and indirectly affect islet *β* cell function through certain mechanisms.

Our study also has some limitations. The sample size was not large enough, the investigation was a single-center study, and there was a low proportion of female patients. Moreover, HOMA-*β* is not a very good surrogate of pancreatic *β* cell function, while direct measures, such as hyperglycemic clamp and acute insulin response, are better methods to assess *β* cell function. Therefore, further research should be carried out to confirm the results of the study.

The findings of this study provide a reference for clinicians to focus on the protection of islet *β* cell function and avoid the application of insulin secretagogues in CHB patients with abnormal glucose metabolism. Future research should be carried out to verify the compromised islet *β* cell function and then to investigate the mechanisms behind the effect of hepatitis B virus infection on islet *β* cell function in CHB patients.

## Figures and Tables

**Table 1:** Baseline comparison between the two groups (*n* = 220).

Variables	CHB group (*n* = 110)	Non-HBV group (*n* = 110)	*t* score or χ^*2*^ score	*P* score
Age (years)	43.86 ± 14.38	42.68 ± 13.34	*t* = 0.794	0.428
Male (number, %)	90 (81.92%)	90 (81.92%)	χ^*2*^ = 0.000	1.000
BMI (kg/m^2^)	22.52 ± 2.74	23.14 ± 4.07	*t* = −1.245	0.215
Glucose metabolism conditions			χ^*2*^ = 0.000	1.000
NGT	50 (45.46%)	50 (45.46%)		
IGR	30 (27.27%)	30 (27.27%)		
DM	30 (27.27%)	30 (27.27%)		

CHB, chronic hepatitis B; non-HBV, without hepatitis B virus infection; BMI, body mass index; NGT, normal glucose tolerance; IGR, impaired glucose regulation; DM, diabetes mellitus.

**Table 2:** Comparison of glucose metabolism conditions between non-LC subgroup and LC subgroup (*n* = 110) (case, %).

Variables	Non-LC subgroup (*n* = 69)	LC subgroup (*n* = 41)	*χ*^2^ score	*P* score
Glucose metabolism conditions			−3.588	<0.001
NGT	42 (60.87)	8 (19.51)		
IGR	12 (17.39)	18 (43.90)		
DM	15 (21.74)	15 (36.59)		

LC, liver cirrhosis; NGT, normal glucose tolerance; IGR, impaired glucose regulation; DM, diabetes mellitus.

**Table 3:** Comparison of glucose metabolism conditions between HBeAg (+) subgroup and HBeAg (−) subgroup (*n* = 110) (case, %).

Variables	HBeAg (+) subgroup (*n* = 38)	HBeAg (−) subgroup (*n* = 72)	*χ*^2^ score	*P* score
Glucose metabolism conditions			−6.174	<0.001
NGT	26 (68.42)	24 (33.33)		
IGR	7 (18.42)	23 (31.94)		
DM	5 (13.16)	25 (34.72)		

NGT, normal glucose tolerance; IGR, impaired glucose regulation; DM, diabetes mellitus.

**Table 4:** Spearman correlation analysis between glucose metabolism parameters and HBV-related biological and serum parameters (*n* = 110).

Variable	Glucose metabolism (1 = NGT, 2 = IGR, and 3 = DM)		FPG (mmol/L)		FINS (mU/L)		HOMA-IR (mU ^*∗*^ mmol/L^2^)		HOMA-*β* (mU/mmol)	
	*r*	*P*	*r*	*P*	*r*	*P*	*r*	*P*	*r*	*P*
Cirrhosis(1 = without, 2 = with)	0.191	0.046			0.322	0.001	0.328	<0.0001		
HBeAg (1 = negative, 2 = positive)	−0.330	<0.0001	−0.268	0.005						
HBsAg	−0.350	<0.0001	−0.334	<0.0001					0.200	0.036
HBsAb					−0.270	0.004	−0.199	0.037	−0.281	0.003
HBeAg	−0.321	0.001	−0.232	0.015						
HBeAb	0.396	<0.0001	0.333	<0.0001			0.240	0.012		
HBcAb	−0.205	0.032								
HBVDNA	−0.202	0.034	−0.190	0.047						
ALP	0.247	0.009	0.286	0.002					−0.361	<0.0001
GGT	0.354	<0.0001	0.293	0.002						
LSM	0.260	0.006	0.272	0.004	0.230	0.015	0.306	<0.0001		

ALP, alkaline phosphatase; FINS, fasting serum insulin; DM, diabetes mellitus; FPG, fasting plasma glucose; GGT, gamma glutamyl transferase; HbA1c, glycosylated hemoglobin; HBcAb, hepatitis B core antibody; HBeAb, hepatitis B envelope antibody; HBeAg, hepatitis B envelope antigen; HBsAb, hepatitis B surface antibody; HBsAg, hepatitis B surface antigen; HBVDNA, hepatitis B viral nucleic acid load; HOMA-*β*, homeostasis model assessment of *β* cells; HOMA-IR, homeostasis model assessment of insulin resistance; IGR, impaired glucose regulation; LSM, liver stiffness measurement; NGT, normal glucose tolerance.

**Table 5:** Multiple stepwise regression analysis of influencing factors of HBV-related biological and serum parameters on glucose metabolism parameters (*n* = 110).

Independent variable		*B*	Std. error	Beta	*t*	*P*
Glucose metabolism (1 = NGT, 2 = IGR, and 3 = DM)	Constant	0.730	0.273	—	2.678	0.009
	HBeAg (1 = negative, 2 = positive)	−0.430	0.161	−0.249	−2.663	0.009
	GGT	0.002	0.001	0.298	3.291	0.001
	HBeAb	0.117	0.045	0.242	2.600	0.011
FPG (mmol/L)	Constant	5.463	0.266	—	20.530	<0.0001
	HBeAb	0.494	0.126	0.378	3.933	<0.0001
FINS (mU/L)	Constant	3.069	0.787	—	3.900	<0.0001
	Cirrhosis (1 = without, 2 = with)	1.123	0.531	0.214	2.117	0.037
HOMA-IR (mU ^*∗*^ mmol/L^2^)	Constant	0.560	0.269	—	2.081	0.040
	HBeAb	0.133	0.054	0.225	2.453	0.016
	Cirrhosis (1 = without, 2 = with)	0.426	0.180	0.216	2.358	0.020
HOMA-*β* (mU/mmol)	Constant	66.452	8.387	—	7.923	<0.0001
	ALP	−0.183	0.074	−0.230	−2.459	0.016

ALP, alkaline phosphatase; FINS, fasting serum insulin; DM, diabetes mellitus; FPG, fasting plasma glucose; GGT, gamma glutamyl transferase; HBeAb, hepatitis B envelope antibody; HBeAg, hepatitis B envelope antigen; HOMA-*β*, homeostasis model assessment of *β* cells; HOMA-IR, homeostasis model assessment of insulin resistance; IGR, impaired glucose regulation; NGT, normal glucose tolerance.

## Data Availability

All the data, models, or codes generated or used during the study are available from Dafeng Liu (e-mail: liudf312@126.com) by request.
